# A meta-analysis of the efficacy and safety of Janus kinase inhibitors in patients with ulcerative colitis

**DOI:** 10.1097/MD.0000000000048637

**Published:** 2026-05-08

**Authors:** Qingqing Yang, Yuerong Yan

**Affiliations:** a Department of Gastroenterology, Jiashan County Second People’s Hospital, Jiashan County, Zhejiang Province, China; b Department of Gastroenterology, Xiangyang Central Hospital, Affiliated Hospital of Hubei University of Arts and Science, Xiangyang City, Hubei Province, China.

**Keywords:** Janus kinase inhibitors, meta-analysis, randomized controlled trials, tofacitinib, ulcerative colitis, upadacitinib

## Abstract

**Background:**

Ulcerative colitis (UC) is a chronic inflammatory bowel disease characterized by relapsing intestinal inflammation and substantial impairment of quality of life. Janus kinase (JAK) inhibitors are orally administered small-molecule agents that interfere with intracellular cytokine signaling pathways central to UC pathogenesis. This systematic review and meta-analysis aimed to assess the therapeutic benefits and safety profile of JAK inhibitors in adults with moderate-to-severe UC.

**Methods:**

A systematic literature search was conducted in PubMed (from inception to November 2025), Embase, the Cochrane Library, and Web of Science to identify placebo-controlled RCTs evaluating JAK inhibitors in adult patients with UC. The primary efficacy outcomes were clinical remission and clinical response. Secondary outcomes included endoscopic remission, endoscopic response, and mucosal healing. Safety was assessed by the incidence of any adverse events (AEs). Pooled risk ratios (RRs) with 95% confidence intervals (CIs) were calculated using fixed- or random-effects models as appropriate. Statistical heterogeneity was evaluated using the *I*^2^ statistic, and subgroup analyses were performed according to treatment phase.

**Results:**

Nine publications encompassing 14 placebo-controlled RCT datasets met the inclusion criteria. Compared with placebo, JAK inhibitors significantly increased rates of clinical remission (RR = 2.48, 95% CI: 1.64–3.73) and clinical response (RR = 2.53, 95% CI: 1.73–3.70), although moderate-to-high heterogeneity was observed. Endoscopic outcomes were also markedly improved, with higher rates of endoscopic remission (RR = 3.52, 95% CI: 2.55–4.86) and mucosal healing (RR = 2.60–2.79 across models). Safety analyses demonstrated no significant difference in the overall incidence of adverse events between JAK inhibitors and placebo. Subgroup analyses revealed consistent efficacy during both induction and maintenance phases, with comparable safety profiles across treatment periods.

**Conclusion:**

JAK inhibitors provide significant improvements in both clinical and endoscopic outcomes for patients with moderate-to-severe UC without increasing the overall risk of adverse events in short-term trials. These findings support the clinical utility of JAK inhibitors in UC management, while highlighting the need for long-term studies to better characterize rare and delayed safety signals.

## 1. Introduction

Ulcerative colitis (UC) is a relapsing–remitting inflammatory bowel disease characterized by continuous colonic mucosal inflammation and symptoms such as diarrhea, rectal bleeding, urgency, and abdominal pain. Conventional therapies (e.g., 5-aminosalicylates, corticosteroids, immunomodulators) and advanced therapies (biologics and small molecules) have improved disease control, but a substantial proportion of patients experience primary nonresponse, loss of response, or intolerance.^[1-4]^

The JAK–STAT pathway mediates signaling from multiple cytokines implicated in UC pathogenesis. JAK inhibitors, as oral small molecules, can inhibit one or more JAK isoforms and thereby attenuate inflammatory signaling while offering practical advantages (oral administration and rapid onset) compared with injectable biologics. Recent comparative evidence and network meta-analyses highlight strong efficacy signals for JAK inhibitors – particularly for rapid symptom relief and induction of remission – although safety remains an essential determinant of treatment positioning.^[5-7]^

Therefore, we performed a systematic review and meta-analysis of RCTs to evaluate the efficacy and safety of JAK inhibitors versus placebo in moderate-to-severe UC.

## 2. Materials and methods

### 2.1. Search strategy and eligibility criteria

This study is a meta-analysis and does not require approval from the ethics committee. This systematic review and meta-analysis was conducted in accordance with the PRISMA (preferred reporting items for systematic reviews and meta-analyses) 2020 statement. We systematically searched MEDLINE (via PubMed), Embase, Web of Science, and the Cochrane Central Register of Controlled Trials (CENTRAL) from inception to November 2025. The search strategy combined Medical Subject Headings (MeSH) and free-text terms related to ulcerative colitis and JAK inhibition, including: “ulcerative colitis,” “Janus kinase inhibitor,” “JAK inhibitor,” “tofacitinib,” “upadacitinib,” “filgotinib,” “JAK-STAT,” “randomized controlled trial,” and “clinical outcome,” with Boolean operators (AND/OR) applied as appropriate. The reference lists of included studies and relevant systematic reviews were manually screened to identify additional eligible publications. Studies were considered eligible if they were randomized controlled trials (RCTs) enrolling adult patients with ulcerative colitis and comparing JAK inhibitors with placebo or standard care. Although observational studies were initially considered, no eligible real-world studies with sufficient data were identified for inclusion in the quantitative synthesis. Therefore, the present analysis was restricted to RCTs.

### 2.2. Trial selection criteria

Studies were considered eligible if they met all of the following criteria:

1. were randomized controlled trials (RCTs);
2. enrolled adult patients (age ≥ 18 years) with ulcerative colitis;
3. compared JAK inhibitors at market-approved doses with each other or with a control group (defined as placebo, no treatment, or standard-of-care comparator, when applicable);
4. reported at least one predefined endpoint for clinical remission, clinical response, endoscopic remission, mucosal healing, change from baseline in Mayo score, and/or safety outcomes including adverse events (AEs) or withdrawals/discontinuations due to adverse events (WDAEs); and
5. were published in English.

Studies were excluded if they:

1. were duplicate publications or reported overlapping cohorts (in which case the most complete/updated report was retained);
2. included animal or in vitro experiments;
3. were published in a language other than English;
4. were case reports, reviews, meta-analyses, editorials/letters, or conference abstracts without sufficient extractable data; or
5. did not contain adequate data on efficacy and safety for inclusion.

If both RCTs and real-world studies were included, we planned primary pooled analyses in RCTs and used observational data for supportive subgroup/sensitivity analyses.

### 2.3. Data extraction and quality assessment

Two researchers independently screened records by title/abstract and then full text. Study eligibility was determined by consensus; if disagreements persisted, an independent expert adjudicated.

For each included study, the following information was extracted using a standardized form: first author, year of publication, study design, underlying condition, number of participants, study duration, population characteristics (e.g., disease severity, prior biologic exposure when available), exposure definition (drug, dose, and treatment phase/duration), concomitant treatments permitted, and all reported outcomes. Different dosages or regimens of the same drug were treated as separate intervention arms.

Risk of bias was assessed independently by 2 reviewers. For RCTs, we used the Cochrane Risk of Bias 2.0 tool (Cochrane, London, UK); for observational studies, we applied the Newcastle–Ottawa Scale (NOS; scores ≥ 7 were considered high quality).

### 2.4. Outcomes and endpoint definitions

Primary efficacy: Clinical remission (defined as Mayo score ≤ 2 with stool frequency score ≤ 1, rectal bleeding score = 0, and endoscopic subscore ≤ 1 without friability).

Secondary efficacy: Clinical response (≥3-point reduction in Mayo score with ≥ 1-point reduction in rectal bleeding or stool frequency), endoscopic remission (endoscopic subscore ≤ 1), and mucosal healing (endoscopic subscore = 0).

Safety: Any adverse events (AEs) infections (including herpes zoster), venous thromboembolic events (VTE), major adverse cardiovascular events (MACE), and treatment discontinuation due to AEs.

### 2.5. Statistical analysis

Meta-analyses were performed using Review Manager (RevMan) version 5.4 (Cochrane, London, UK) and Stata version 17.0 (StataCorp LLC, College Station). Dichotomous outcomes were summarized as risk ratios (RRs) with 95% confidence intervals (CIs); continuous outcomes were summarized as mean differences (MDs) with 95% CIs, when applicable.

Statistical heterogeneity was evaluated using the *I*^2^ statistic and interpreted as low (*I*^2^ < 50%), moderate (50–75%), or high (≥75%). A fixed-effects model was used when heterogeneity was low; otherwise, a random-effects model was applied. Prespecified subgroup analyses were conducted by agent (tofacitinib, upadacitinib, filgotinib)where data permitted. Publication bias was assessed by funnel plots and Egger test when ≥ 10 studies were available for an outcome (*P* < .05 indicating potential small-study effects). Sensitivity analyses were conducted by sequentially excluding each study to evaluate robustness.

## 3. Results

### 3.1. Study selection

**Figure 1.**
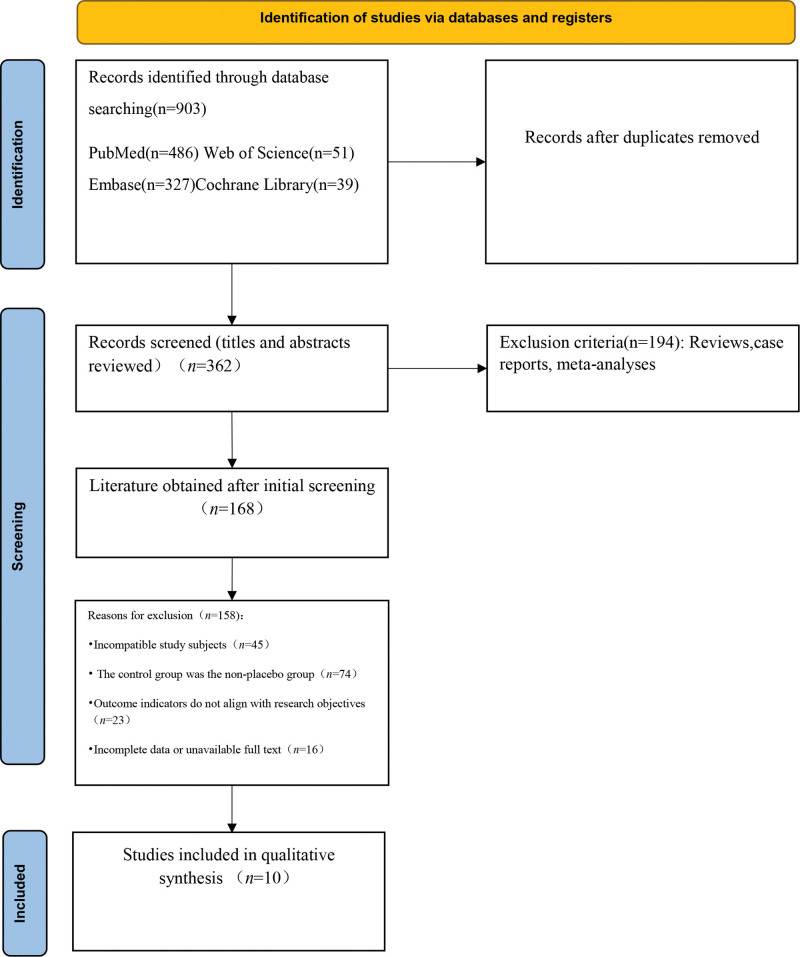
PRISMA flow chart for study selection process. PRISMA = preferred reporting items for systematic reviews and meta-analyses.

The database search identified 903 potentially relevant records. After removal of 447 duplicates, 456 records remained. Of these, 362 records were screened by titles and abstracts, and 194 were excluded as reviews, case reports, or meta-analyses. The full texts of the remaining 168 articles were assessed for eligibility). Ultimately, 9 studies were included in the qualitative synthesis, and 14 randomized controlled trials (RCTs) were eligible for and included in the quantitative meta-analysis (Fig. 1).

### 3.2. Characteristics of included studies

All included studies were randomized, double-blind, placebo-controlled trials enrolling adults with moderate-to-severe ulcerative colitis, with a total sample size of 6925 participants (Table 1). The evidence base comprised 9 publications including 14 trial datasets, of which 8 evaluated induction therapy (approximately 8–11 weeks) and 6 evaluated longer-term/maintenance treatment (approximately 47–58 weeks). The investigated JAK inhibitors included tofacitinib (3 studies) and upadacitinib (2 studies), filgotinib (3 studies), peficitinib (1 study), with different dose arms compared against placebo. Across trials, outcomes commonly reported clinical response, clinical remission, and safety endpoints (including adverse events and serious adverse events).

**Table 1:** The basic features of studies included in the meta-analysis.

Included study	Country/region	Study type	Observation period	Intervention (experimental group/control group)	Sample size (experimental group/control group)	Outcome
Sands et al^[8]^	Global multicenter (including Europe, America, etc)	Double-blind RCT	8 wk (induction phase)	Experimental: Peficitinib 25 mg QD, 75 mg QD, 150 mg QD, 75 mg BID; control: placebo	25 mg group: 44/75 mg QD group: 44/150 mg QD group: 44/75 mg BID group: 44; control group: 43	1, 2, 3, 4, 5
Sandborn et al^[9]^	28 countries (including the United States, Japan, South Korea, Germany, etc)	Double-blind RCT	8 wk (induction phase)	Experimental: Upadacitinib 7.5 mg QD, 15 mg QD, 30 mg QD, 45 mg QD; control: placebo	7.5 mg QD group: 47/15 mg QD group: 49/30 mg QD group: 52/45 mg QD group: 56; control group: 46	1, 2, 3, 4, 5
Feagan et al^[10]^	40 countries (including America, Europe, Asia, Australia, etc)	Double-blind RCT	10 wk (induction phase)	Experimental: Filgotinib 100 mg QD, 200 mg QD; control: placebo	100 mg group: 277/200 mg group: 245; control group: 137	1, 2, 3, 4, 5
Feagan et al^[10]^	40 countries (including America, Europe, Asia, Australia, etc)	Double-blind RCT	10 wk (induction phase)	Experimental: Filgotinib 100 mg QD, 200 mg QD; control: placebo	100 mg group: 285/200 mg group: 262; control group: 142	1, 2, 3, 4, 5
Feagan et al^[10]^	40 countries (including America, Europe, Asia, Australia, etc)	Double-blind RCT	58 wk	Experimental: Filgotinib 100 mg QD, 200 mg QD; control: placebo	100 mg group: 179/200 mg group: 202; control group: 190 (100 mg corresponding: 91/200 mg corresponding: 99)	1, 2, 3, 4, 5
Danese et al (UC1)^[11]^	39 countries (including Europe, America, Asia, Africa, Australia, etc)	Double-blind RCT	8 wk (induction phase)	Experimental: Upadacitinib 45 mg QD; control: placebo	Experimental group: 319; control group: 155	1, 2, 3, 4, 5
Danese et al (UC2)^[11]^	40 countries	Double-blind RCT	8 wk (induction phase)	Experimental: Upadacitinib 45 mg QD; control: placebo	Experimental group: 345; control group: 177	1, 2, 3, 4, 5
Danese et al^[11]^	35 countries	Double-blind RCT	52 wk	Experimental: Upadacitinib 15 mg QD, 30 mg QD; control: placebo	15 mg group: 148/30 mg group: 154; control group: 149	1, 2, 3, 4, 5
Sandborn et al^[12]^	Global multicenter (including Europe, America, Asia, etc)	Double-blind RCT	8 wk (induction phase)	Experimental: Tofacitinib 10 mg BID; control: placebo	Experimental group: 905; control group: 234	1, 2, 3, 4, 5
Mukherjeeet al^[13]^	Global multicenter (including Europe, America, Asia, etc)	Double-blind RCT	52 wk	Experimental: Tofacitinib 5 mg BID, 10 mg BID; control: placebo	5 mg group: 198/ 10 mg group: 196; control group: 198	1, 3
Hibi et al^[14]^	Japan	Double-blind RCT	47 wk	Experimental: Filgotinib 100 mg QD, 200 mg QD; control: placebo	100 mg group: 14/200 mg group: 20; control group: 20	1, 2, 3, 4, 5
Schreiber et al^[15]^	Global multicenter (including Europe, America, Asia, etc)	Double-blind RCT	48 wk	Experimental: Filgotinib 200 mg QD; control: placebo	200 mg group: 199; control group: 98	4
Schreiber et al^[15]^	Global multicenter (including Europe, America, Asia, etc)	Double-blind RCT	11 wk (induction phase)	Experimental: Filgotinib 200 mg QD; control: placebo	200 mg group: 505; control group: 277	4
Lee et al^[16^^]^	Global multicenter (including Europe, America, Asia, etc)	Post hoc stratified analysis of double-blind RCT	52 wk	Experimental: Tofacitinib 5 mg BID, 10 mg BID; control: placebo	5 mg group: 83/10 mg group: 68; control group: 75	1, 2, 3, 4, 5

Outcome: 1 = clinical remission, 2 = clinical response, 3 = endoscopic remission, 4 = mucosal healing, 5 = adverse events.

Standardized English terms for study designs (RCT = randomized controlled trial), administration frequencies (QD = once daily, BID = twice daily).

BID = bis in die (twice daily), QD = quaque die (once daily), RCT = randomized controlled trial.

### 3.3. Risk of bias assessment

**Figure 2.**
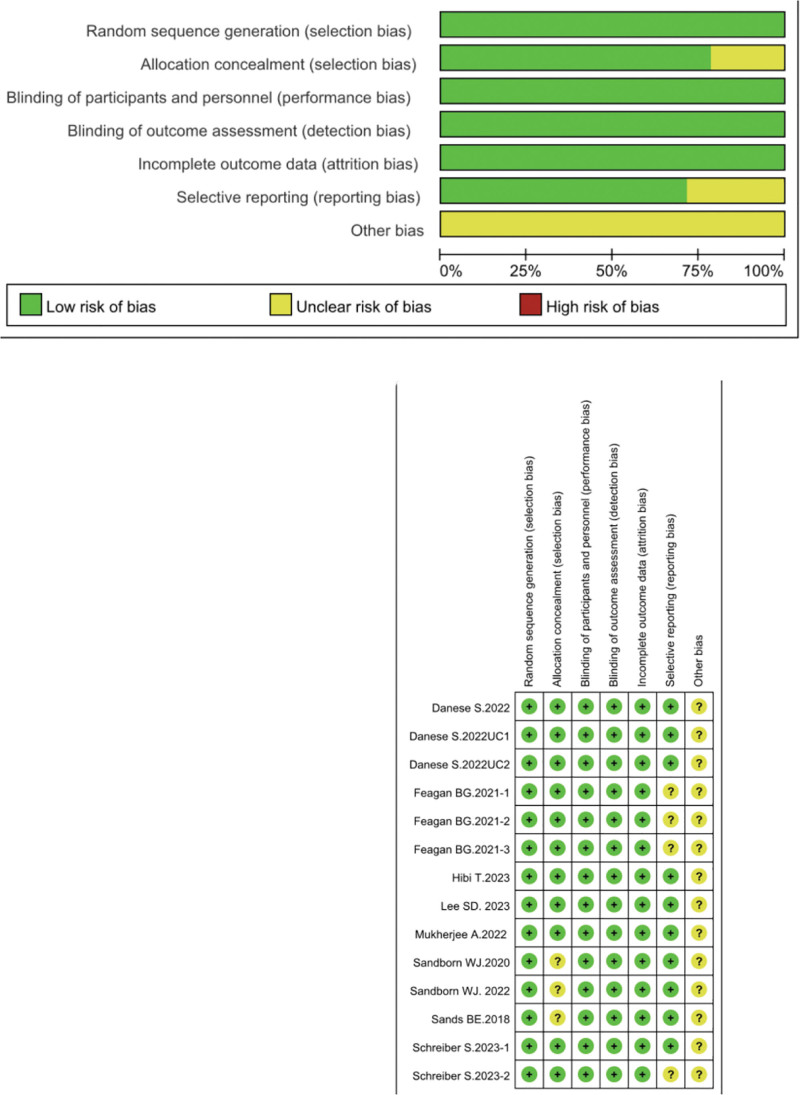
Inclusion of literature quality assessment.

Risk of bias was evaluated using the Cochrane Risk of Bias Tool 2.0. Overall, all included 14 trials were judged to be of high quality, with most trials showing a low risk of bias across all key domains (including random sequence generation, allocation concealment, blinding of participants and personnel, blinding of outcome assessment, incomplete outcome data, and selective reporting). Detailed risk-of-bias assessments for each trial are provided in Figure 2.

### 3.4. Efficacy outcomes

#### 3.4.1. Clinical remission

**Figure 3.**
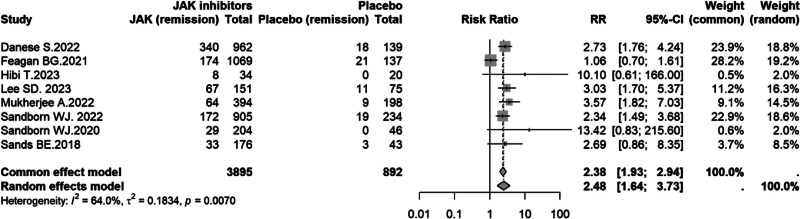
Forest plot of clinical remission comparing JAK inhibitors versus placebo in UC. CI = confidence interval, JAK = Janus kinase, RR = risk ratio, UC = ulcerative colitis.

Eight studies^[8–14,16]^ evaluating 4 JAK inhibitors were included in the analysis of clinical remission. Moderate heterogeneity was observed (*I*^2^ = 64.0%, *P* = .007); therefore, a random-effects model was applied. Overall, treatment with JAK inhibitors was associated with a significantly higher clinical remission rate compared with placebo (RR = 2.48, 95% CI: 1.64–3.73; Fig. 3).

#### 3.4.2. Clinical response

**Figure 4.**
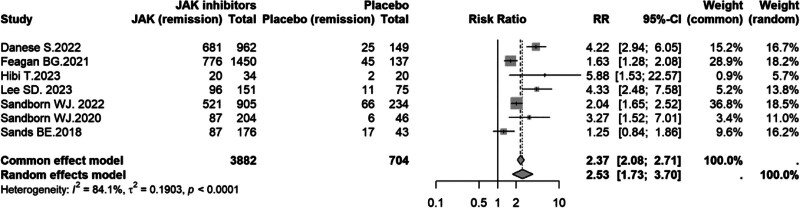
Forest plot of clinical response comparing JAK inhibitors versus placebo in UC. CI = confidence interval, JAK = Janus kinase, RR = risk ratio, UC = ulcerative colitis.

Seven studies^[8-12,14,16]^ reported clinical response during induction therapy. Substantial heterogeneity was detected across studies (*I*^2^ = 84.1%, *P* < .0001), and a random-effects model was used. JAK inhibitors significantly improved clinical response compared with placebo (RR = 2.53, 95% CI: 1.73–3.70; Fig. 4).

#### 3.4.3. Endoscopic remission

**Figure 5.**
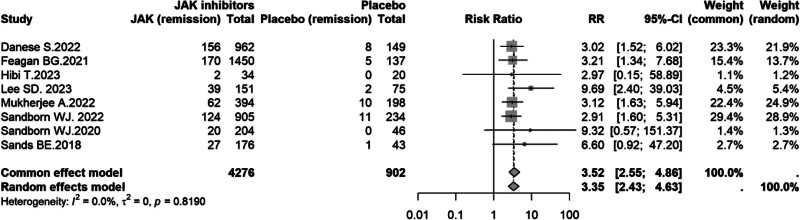
Forest plot of endoscopic remission comparing JAK inhibitors versus placebo in UC. CI = confidence interval, JAK = Janus kinase, RR = risk ratio, UC = ulcerative colitis.

Eight studies^[8–14,16]^ were included to assess endoscopic remission during induction. No heterogeneity was detected (*I*^2^ = 0.0%, *P* = .819); thus, a fixed-effect (common-effect) model was used. JAK inhibitors significantly increased endoscopic remission compared with placebo, with an approximately threefold improvement (RR = 3.52, 95% CI: 2.55–4.86; Fig. 5).

#### 3.4.4. Mucosal healing

**Figure 6.**
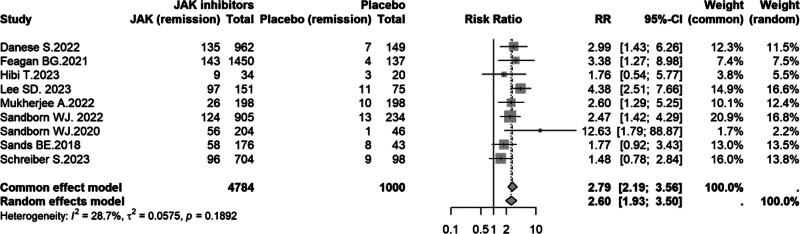
Forest plot of mucosal healing comparing JAK inhibitors versus placebo in UC. CI = confidence interval, JAK = Janus kinase, RR = risk ratio, UC = ulcerative colitis.

Nine trials^[8–16]^ were included in the analysis of mucosal healing during induction. Heterogeneity was low (*I*^2^ = 28.7%, *P* = .1892); therefore, a fixed-effect model was applied. JAK inhibitors significantly improved mucosal healing compared with placebo (fixed-effect model: RR = 2.79, 95% CI: 2.19–3.56). The random-effects estimate was consistent (RR = 2.60, 95% CI: 1.93–3.50), supporting a robust treatment benefit (Fig. 6).

### 3.5. Safety outcomes

**Figure 7.**
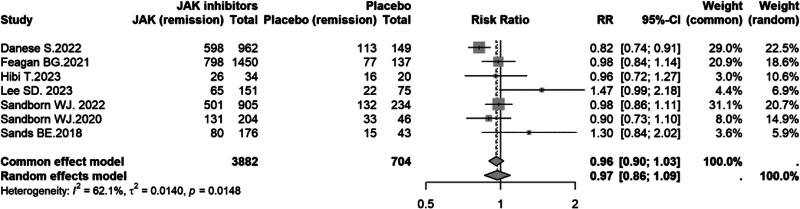
Forest plot of AEs comparing JAK inhibitors versus placebo in UC. AE = adverse event, CI = confidence interval, JAK = Janus kinase, RR = risk ratio, UC = ulcerative colitis.

Seven studies^[8-12,14,16]^ compared the incidence of any adverse events (AEs) between JAK inhibitors and placebo. Moderate heterogeneity was observed (*I*^2^ = 62.1%, *P* = .0148), and a random-effects model was used. There was no statistically significant difference in AE rates between groups (fixed-effect model: RR = 0.96, 95% CI: 0.90–1.03; random-effects model: RR = 0.97, 95% CI: 0.86–1.09), as both confidence intervals crossed unity (Fig. 7).

### 3.6. Subgroup analyses

#### 3.6.1. Clinical remission by treatment phase (induction vs maintenance)

**Figure 8.**
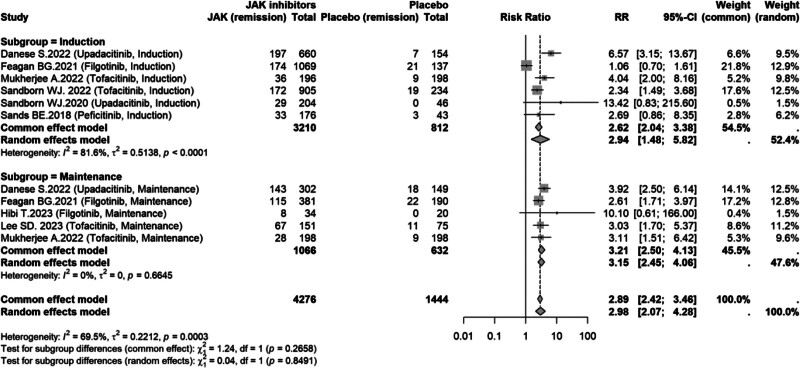
Subgroup analysis of clinical remission by treatment phase (induction vs maintenance). CI = confidence interval, JAK = Janus kinase, RR = risk ratio.

In the phase-stratified subgroup analysis of clinical remission, JAK inhibitors significantly increased the likelihood of achieving remission compared with placebo in both the induction phase (RR = 2.94, 95% CI: 1.48–5.82; substantial heterogeneity, *I*^2^ = 81.6%) and the maintenance phase (RR = 3.15, 95% CI: 2.45–4.06; *I*^2^ = 0%). Overall, JAK inhibitor therapy remained associated with a markedly higher clinical remission rate (RR = 2.98, 95% CI: 2.07–4.28; *I*^2^ = 69.5%). The test for subgroup differences indicated no significant interaction between treatment phase and effect size under the random-effects model (*P* = .8491; Fig. 8).

#### 3.6.2. Adverse events by treatment phase (induction vs maintenance)

**Figure 9.**
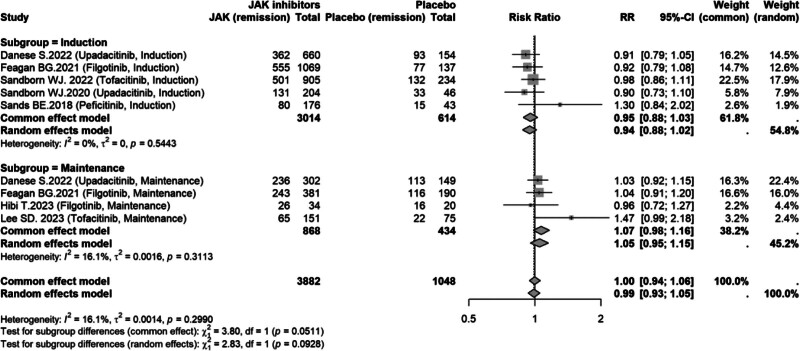
Subgroup analysis of AEs by treatment phase (induction vs maintenance). AE = adverse event, CI = confidence interval, JAK = Janus kinase, RR = risk ratio.

For the AE rate, subgroup analyses showed no significant difference between JAK inhibitors and placebo in either the induction phase (RR = 0.94, 95% CI: 0.88–1.02; *I*^2^ = 0%) or the maintenance phase (RR = 1.05, 95% CI: 0.95–1.15; *I*^2^ = 16.1%). The pooled estimate across phases similarly suggested comparable AE risk (RR = 0.99, 95% CI: 0.93–1.05; *I*^2^ = 16.1%). Consistent with these findings, the subgroup-difference test did not demonstrate a statistically significant phase effect (*P* = .0928, random-effects; Fig. 9).

## 4. Discussion

### 4.1. Principal findings and clinical implications

Ulcerative colitis is a chronic relapsing–remitting inflammatory bowel disease in which the core therapeutic goals are to control disease activity, induce and maintain remission, alleviate symptom burden, and ultimately improve health-related quality of life.^[15,17]^ Over the past 2 decades, the introduction of biologics – particularly anti-tumor necrosis factor (TNF) agents and newer mechanism-based therapies such as anti-IL-12/23 (interleukin-12/23) – has substantially expanded treatment options and reshaped UC management.^[18-20]^ Nevertheless, important unmet needs remain in routine practice: a meaningful proportion of patients experience primary nonresponse or loss of response to biologics, and long-term treatment can be constrained by cost, route of administration, safety concerns, and treatment persistence.

Against this background, oral JAK inhibitors represent an attractive strategy because they target the JAK/STAT pathway, a central signaling hub for multiple cytokines implicated in both innate and adaptive immune activation in UC.^[21–23]^ By inhibiting JAK-mediated cytokine signaling, these small molecules can attenuate inflammatory cascades and mucosal injury, providing a mechanistic rationale for broad anti-inflammatory efficacy across clinical and endoscopic targets.^[24]^

This meta-analysis comprehensively compared the efficacy and safety of 5 JAK inhibitors (tofacitinib, upadacitinib, filgotinib, peficitinib) and placebo in patients with moderate-to-severe UC, integrating data from 9 studies involving 15 RCTs. Consistent with prior evidence syntheses, our results confirm that JAK inhibitor therapy significantly improves key treat-to-target outcomes compared to placebo, including clinical remission, clinical response, endoscopic remission, and mucosal healing – core endpoints reflecting disease control and long-term prognosis in UC.

### 4.2. Interpretation of heterogeneity and subgroup findings

We observed substantial heterogeneity for some efficacy outcomes (notably clinical response), which is unsurprising given differences across trials in the specific JAK inhibitor evaluated (with varying isoform selectivity), study phase (induction vs maintenance), dosing strategies and background therapies, and baseline patient features such as disease severity and prior biologic exposure. These clinical differences can translate into variability in absolute placebo response, timing of assessment, and responsiveness to pathway inhibition, thereby inflating between-study heterogeneity.

Importantly, phase-stratified subgroup analyses indicated that the direction of benefit was consistent across induction and maintenance periods, and formal tests did not suggest a significant interaction between phase and treatment effect. This finding is clinically relevant because UC treatment is fundamentally a 2-step strategy (induction then maintenance): therapies that demonstrate both early response and sustained control are particularly valuable for long-term disease management. The observation of low heterogeneity in maintenance analyses may reflect more homogeneous trial designs and more stable response definitions at later time points.

### 4.3. Safety profile and practical considerations

From a safety standpoint, pooled analyses did not show a statistically significant increase in overall adverse events compared with placebo. This is reassuring and aligns with the conceptual advantage of small molecules such as shorter half-life and rapid discontinuation if adverse events occur. However, safety interpretation should remain cautious. RCT follow-up is often insufficient to fully quantify uncommon or long-latency outcomes, and adverse events of special interest for the JAK inhibitor class (e.g., certain infections such as herpes zoster, thromboembolic events, and cardiovascular events) may require larger datasets and longer observation to characterize precisely.

### 4.4. Limitations

Several limitations should be acknowledged. First, the number of eligible placebo-controlled RCT datasets remains limited for some endpoints, which restricts statistical power for rare harms and limits the precision of subgroup analyses. Second, variability in trial design (induction vs maintenance), dosing regimens, and background therapy may contribute to heterogeneity and complicate direct cross-trial comparability. Third, patient-level factors such as prior biologic exposure, concomitant steroid use, and baseline disease severity were not consistently reported, precluding robust stratified analyses. Fourth, our analysis did not evaluate cost-effectiveness, which is a key determinant of real-world treatment choice given the economic burden of advanced UC therapies. Finally, if treatment ranking methods are applied, they should be interpreted cautiously because rankings may not fully capture the magnitude of clinical benefit, uncertainty in estimates, or differences in follow-up duration and safety ascertainment. The safety findings should be interpreted with caution, as most included trials had relatively short follow-up durations and were not powered to detect rare or long-term adverse events.

### 4.5. Future directions

Future research should prioritize long-term real-world studies to evaluate durability of response, treatment persistence, and late-onset adverse outcomes across diverse populations, including patients with comorbidities and those with prior biologic failure. Head-to-head comparative studies and well-designed network meta-analyses with consistent endpoint definitions may further clarify relative effectiveness among different advanced therapies and among JAK inhibitors with different selectivity profiles. In addition, pharmacoeconomic evaluations are needed to inform payers and clinicians about value-based positioning of oral small molecules versus biologics. Finally, the potential role of JAK inhibitors in high-risk subgroups – such as acute severe UC requiring hospitalization – merits focused investigation to determine whether rapid anti-inflammatory effects translate into reduced colectomy risk and improved short-term outcomes.

## 5. Conclusion

In summary, this meta-analysis indicates that JAK inhibitors provide significant improvements in clinical and endoscopic outcomes in adults with moderate-to-severe UC compared with placebo, with no clear increase in overall adverse events in trial settings. These findings support JAK inhibitors as effective oral advanced therapies, while emphasizing the need for ongoing safety surveillance and longer-term studies to refine patient selection and optimize treatment algorithms. However, long-term safety remains uncertain and requires further investigation.

## Acknowledgments

The author wishes to express their gratitude to the team for their valuable contributions, help, and insightful discussions throughout the project.

## Author contributions

**Conceptualization:** Qingqing Yang, Yuerong Yan.

**Data curation:** Qingqing Yang, Yuerong Yan.

**Formal analysis:** Qingqing Yang, Yuerong Yan.

**Funding acquisition:** Yuerong Yan.

**Investigation:** Yuerong Yan.

**Writing** – **original draft:** Yuerong Yan.

**Writing** – **review & editing:** Yuerong Yan.
